# Diffusion-Weighted Magnetic Resonance Imaging: Clinical Potential and Applications

**DOI:** 10.3390/jcm11123339

**Published:** 2022-06-10

**Authors:** Anna Caroli

**Affiliations:** Bioengineering Department, Istituto di Ricerche Farmacologiche Mario Negri IRCCS, 24020 Ranica, BG, Italy; acaroli@marionegri.it

Since its discovery in the 1980s [1], diffusion-weighted MRI (DWI), a non-invasive imaging method that does not require any tracer injection, has experienced exponential growth, becoming a pillar of modern clinical imaging.

DWI enables the investigation of tissue microstructure. In fact, during their random, diffusion-driven displacements, water molecules microscopically probe the tissue structure, interacting with cell membranes, thus providing unique information on the tissue’s functional architecture [2].

DWI is based on Einstein’s diffusion equation [3], assuming free diffusion as in a glass of water, with the distribution of molecular displacements obeying a Gaussian law. However, this is not the case in biological tissues since molecular displacements are hindered by many obstacles, such as cell membranes, fibers, or macromolecules. The apparent diffusion coefficient (ADC) concept [1] uses Einstein’s equation to model diffusion MRI signals while emphasizing that the DWI-based diffusion coefficient in the tissue is no longer the free diffusion coefficient of water. ADC, intrinsically reflecting the interaction of water molecules with tissue elements, proved high sensitivity to pathologic or physiologic conditions and is still widely used.

Within each image voxel, different types of incoherent motion, such as blood microcirculation in the capillary networks, on top of molecular diffusion, contribute to DWI signal attenuation [4]. Just as with water molecule displacements, blood flow in randomly oriented capillaries follows a random walk called pseudodiffusion. Since the pseudodiffusion coefficient (D*) is an order of magnitude higher than the water diffusion coefficient (D), true diffusion can be separated from pseudodiffusion through the use of intra-voxel incoherent motion (IVIM) models [5,6], providing information in addition to the summary ADC parameter.

Although diffusion is a 3D process, it is only measured in one dimension at a time. However, in some tissues, such as white matter fibers of the brain, diffusion may be anisotropic, with diffusion effects strongly depending on the gradient pulse directions. Anisotropic diffusion cannot be simply measured along three perpendicular axes; rather, it requires the use of a tensor. Diffusion tensor imaging (DTI) [7] provides information on the main diffusion directions for each image voxel, therefore enabling the assessment of the direction of the tissue fibers, denoted by the fastest diffusion.

Even though its main clinical field of application has historically been neuroscience, where it is extremely useful for investigating neurological disorders such as acute brain ischemia or probing human brain connectivity [8,9,10], in recent years, diffusion MRI has experienced remarkable momentum, rapidly becoming an imaging modality of choice for a variety of other organs.

DWI has been widely used in cancer imaging to detect, characterize, and stage malignant lesions, or even assess the response to therapy, in various anatomical districts besides the brain [11], such as the breast [12,13], lung [14,15], liver [16], pancreas [17], kidney [18], gallbladder [19], thyroid [20], head and neck [21,22], prostate [23,24], vertebral bone marrow [25], musculoskeletal soft tissue [26], nasopharynx [27], ovaries [28], cervix [29], and rectum [30], as well as in the whole body [31].

DWI and DTI have also seen a wide spectrum of non-oncological applications throughout the body, including but not limited to the kidney [32], liver [33,34], and breast [35]. DWI has proven promising to detect and stage fibrosis [36,37] as well as to characterize inflammation [38,39]. Moreover, thanks to its non-invasiveness, DWI has been widely adopted in the pediatric population [40,41,42,43,44,45,46,47].

The collection of articles published in this Special Issue further highlights the clinical potential of DWI in the brain and beyond. A few review papers on the genito-urinary system [48], ovarian cancer [49], and non-myelopathic cervical spinal cord compression [50] are included to complement what has been published so far.

Several original papers explore novel DWI applications in different clinical contexts. Ota and colleagues [51] investigated the capability of diffusion-based virtual MR elastography (VMRE) in the characterization of liver tumors, finding that its combination with MR elastography, and therefore the combination of stiffness and microstructure information, improved discriminant performance between hepatocellular carcinoma and metastases. Thiel and colleagues [52] showed DWI/DTI’s potential to identify patients at risk for contrast-induced nephropathy development shortly after iodine-containing X-ray contrast medium administration. Diffusion MRI, reflecting acute changes in the renal microstructure even before alterations in laboratory parameters, could become part of a multi-modality-based clinical protocol alongside clinical information, routine renal parameters, and advanced urinary injury markers and may help facilitate the appropriate therapy in at-risk patients. Mani and colleagues [53] used DWI combined with blood-oxygen-level-dependent (BOLD) MRI to non-invasively assess differences in kidney graft diffusion, perfusion, and oxygenation over time between kidney transplant recipients treated with ciclosporin or everolimus. Despite negative preliminary findings due to the limited sample size, the study findings showed an improvement in graft diffusivity over time and a tendency for ameliorated tissue oxygenation after the switch to everolimus, suggesting an impact of the immunosuppressive regimen on fMRI parameters of the kidney graft. Quattrini and colleagues [54] used DTI to detect white matter microstructural impairment of the triple network system in patients with borderline personality disorder, finding more pronounced diffusion alterations in patients with higher behavioral dysregulation. Anfigeno and colleagues [55] provided preliminary evidence in favor of DWI’s potential to non-invasively detect renal parenchymal involvement in pediatric patients with febrile urinary tract infection. DWI showed higher sensitivity to areas of pyelonephritis compared to ultrasound, even if the latter still maintains an important role in the pyelonephritis diagnostic process.

This Special Issue also includes two preclinical contributions. Schmidbauer and colleagues [56] showed the potential of multiparametric MRI—DWI in combination with T1 and T2 mapping—for non-invasive monitoring of long-term changes in renal allografts in a translational mouse model and for guiding invasive assessment by allograft biopsy. The authors also highlighted the added value of combining ADC quantitative assessment with histogram-based analysis, finding an association between evidence of inflammation and interstitial fibrosis in allografts with chronic rejection and increased ADC heterogeneity, likely because of the heterogeneous texture of chronic allograft rejection. Merchant and colleagues [57] highlighted IVIM-DTI’s potential in assessing peripheral nerve injury and regenerative potential in a preclinical rodent model. The authors could separate microcirculation (denoted by a faster-decaying diffusion signal) from tissue microstructure (denoted by a slower-decaying signal, also found in postmortem nerves lacking perfusion) in normal sciatic nerves, highlighting the benefit and need to combine IVIM with DTI to simultaneously but separately characterize the behavior of the two compartments, which are both highly anisotropic.

The paper by Li and colleagues [58] showed the preliminary feasibility and promise of radiomics-based analysis applied to quantitative kidney diffusion-weighted MRI. Radiomics, using specific algorithms to extract features from medical images inherently capturing spatial information, may uncover patterns, textures, or characteristics that may serve as digital fingerprints of disease, in addition to individual DWI biomarkers such as ADC.

Last, the paper by Thieleking and colleagues [59] provided interesting insights into DWI/DTI challenges. Indeed, this inter-site comparability study performed on healthy subjects reported systematic global and regional differences in common DTI outcome measures between different scanners, sequences, and processing pipelines. Since regionally inhomogeneous variations may considerably confound physiological effects and pathological changes, this paper highlights the need to harmonize DWI data acquisition and analysis to augment imaging data reliability and replicability, in particular in the context of multicenter longitudinal clinical studies, and to estimate normative values for clinical practice. Recent DWI harmonization efforts and consensus standardization initiatives in different clinical contexts, such as the spine [60], the kidney [61], the breast [35], and whole-body DWI in cancer [62], have pointed the way forward.

In conclusion, besides illustrating diffusion MRI’s clinical potential in both acute and chronic settings and providing an overview of possible diffusion applications in different clinical contexts, the current Special Issue also highlights cross-cutting challenges of its routine use in clinical practice for diagnosis, disease staging, disease progression monitoring, as well as for potentially guiding tailored therapy management. DWI and DTI, providing valuable qualitative and quantitative information to probe tissue microstructures, should be combined with other imaging modalities in a multiparametric approach to gain the best insight into the pathophysiology. Thanks to its non-invasiveness and versatility, multiparametric MRI seems the best way forward, except for multi-morbid patients with specific contraindications. Its combination with anatomical sequences is needed for the optimal localization of pathological areas; moreover, its combination with other sequences providing complementary information on tissue/organ microstructure and function (such as T1 and T2 mapping, MR elastography, BOLD, or functional MRI) is strongly recommended. Special care should be taken in the acquisition protocol, in particular to fat suppression and the choice of optimal b values. Moreover, since a long acquisition time could be a challenge, both in terms of the availability of the scan time and acceptability by unwell patients, the acquisition time needs to be short enough to be compliant with clinical practice.

Given the potential of DWI as an effective imaging biomarker, its exponential growth is expected to continue over the years, extending to additional clinical contexts, opening new fascinating research avenues, and ultimately increasing its use in clinical practice.
